# Angiogenesis Status in Patients with Acute Myeloid Leukemia: From Diagnosis to Post-hematopoietic Stem Cell Transplantation

**Published:** 2017-05-01

**Authors:** M. Mohammadi Najafabadi, K. Shamsasenjan, P. Akbarzadehalaleh

**Affiliations:** 1 *Immunology Research Center, Tabriz University of Medical Sciences, Tabriz, Iran*; 2 *Department of Pharmaceutical Biotechnology, Faculty of Pharmacy, Tabriz University of Medical Sciences, Tabriz, Iran*

**Keywords:** Leukemia, myeloid, acute, Angiogenesis inducing agents, Vascular endothelial growth factor A, Endothelial cells, Hematopoietic stem cell transplantation

## Abstract

As already proven in solid tumors, increased angiogenesis leads to increased number of blood vessels, resulting in unfavorable outcomes and resistance to chemotherapy. It was previously thought that angiogenesis plays no role in the pathogenesis of acute myeloid leukemia (AML), due to the fact that AML is a liquid tumor. However, many studies have suggested that increased angiogenesis has important roles in patients with AML, including increased numbers of vessels in bone marrow and pro-angiogenic factors, as well as decreased anti-angiogenic factors. Also a large number of studies demonstrated that a two-way communication is established between leukemic and endothelial cells, as a component of the vessel wall, in the bone marrow of patients with AML. These two cells support the survival and proliferation of each other through a paracrine pathway, resulting in resistance to chemotherapy. In addition, It is well-established that increased angiogenesis is associated with unfavorable prognosis, lower survival rate, resistance to chemotherapy, and relapse. Furthermore, increased angiogenesis affects the response to treatment, hematopoietic stem cell transplantation (HSCT) outcome and graft versus host disease (GVHD) occurrence. In this regard, this review will address vascular endothelial growth factor (VEGF) and angiopoietin (Ang), two of the most important angiogenic factors, in patients with AML before and after HSCT. By increasing our understanding of the role of endothelial cells and angiogenic factors in patients with AML from diagnosis to post-HSCT, new therapeutic strategies can be developed to reduce angiogenesis, improve patients’ survival and reduce complications.

## INTRODUCTION

Angiogenesis is the biological process by which new blood vessels develop from pre-existing ones [1] and is the result of balance between pro- and anti-angiogenic factors [2]. This process is essential for tumor growth and development [1]. Many tumors primarily grow along blood vessels until they reach a certain size and then, due to local hypoxia, nutrient depletion, and metabolic imbalance, both tumor cells and the related stromal components produce tumor angiogenic factors (TAFs). Thereafter, additional growth of the tumor depends on the formation of new blood vessels [1, 3]. Acute myeloid leukaemia (AML) is caused by mutations in hematopoietic stem cells and progenitor cells. AML is characterized by accumulation of immature myeloid cells in the bone marrow, which eventually leads to impaired normal hematopoiesis. The symptoms of AML include anemia, thrombocytopenia and increased abnormal white blood cells in the peripheral blood [4]. Via standard chemotherapy regimens, the initial disease remission can be reached in 30%–70% of patients with AML, though in all patients, particularly in older individuals, refractory and relapsed disease remains a main problem [5, 6]. One of the treatments for patients with AML is allogeneic hematopoietic stem cell transplantation (allo-HSCT) [7]. In spite of major advances in this field, yet the treatment is associated with significant problems and late effects. Along with relapses and severe infections, the major reason of mortality and morbidity after allo-HSCT is graft versus host disease (GVHD). When GVHD occurs, it is hard to treat; therefore, prevention of its occurrence and early detection are essential for a favorable outcome [8]. 

Because the bone marrow is the major place of tumor cells in AML, there is no compact well-formed structured tumor. Furthermore, due to the historical beliefs about leukemia and considering it a liquid tumor, it was initially believed that angiogenesis would play only a minor role in the pathogenesis of AML [9]. More recently, researchers have found evidence of increased angiogenesis in patients with leukemia [10]. In addition, endothelial injury and angiogenesis play important roles in the conditions after HSCT and occurrence of GVHD [11].

In this light, in this review we present various aspects of angiogenesis in patients with AML from diagnosis to post-HSCT. To do so, we initially present the role of endothelial cells in patients with AML and the number of vessels in the bone marrow, which is reported as MVD. Then, two of the most important and the main angiogenic factors, VEGF and Ang2, are examined. Also in each section, we assess the relationship between any of these angiogenic factors with the clinical course of the disease.

Two-way Communication between AML Cells and Endothelial Cells

**Figure 1:**
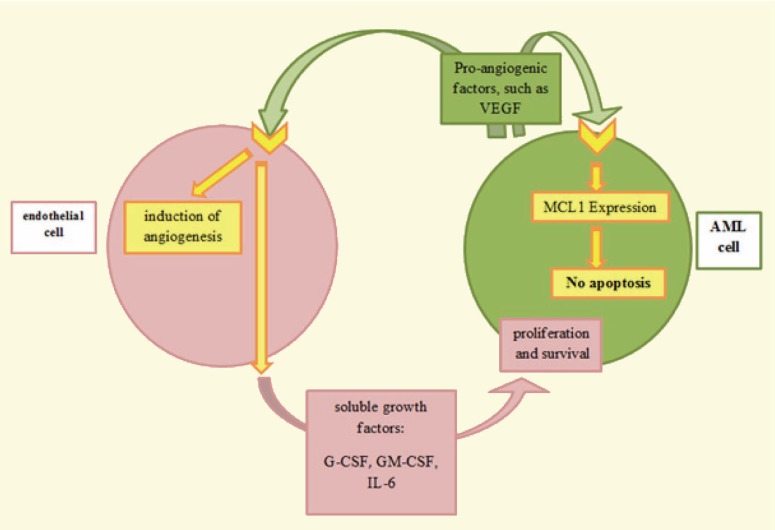
Two-way communication between AML cells and endothelial cells. VEGF: vascular endothelial growth factor; MCL1: myeloid cell leukemia 1; G-CSF: granulocyte colony-stimulating factor; GM-CSF: granulocyte-macrophage colony-stimulating factor; IL-6: interleukin-6

There are two main niches in the bone marrow that play important roles in hematopoiesis: the hypoxic endosteal niche in which hematopoietic stem cells are in contact with osteoblasts, and the vascular niche, with a higher oxygen pressure [12, 13], in which more differentiated hematopoietic cells are in contact with the vascular sinusoids and arterioles [14, 15]. In contrast to other parts of the body, vascular architecture in the bone marrow forms a sinusoidal network [16] consisting of a single layer of endothelial cells (ECs) [17]. Several studies have shown that human bone marrow ECs in culture not only support the proliferation and differentiation of human hematopoietic cells, but also secrete several hematopoietic cytokines [18]. Many studies have also shown that bone marrow vessels, in both healthy and diseased states, play an important role in hematopoiesis through the production of soluble factors and cell-cell contact. In situations where hematopoiesis is impaired including AML, leukemic cells are localized around blood vessels [19], and an increase in sinusoidal blood vessels occurs in the central part of the bone marrow [20]. In this regard Dilly and co-workers have shown that increased amounts of endothelial cells were found in tissue sections taken from bone marrow biopsy of patients with AML [21]. In addition, not only were the numbers of circulating endothelial cells (CECs) higher in the peripheral blood of patients with AML, as compared to the control, but also their levels were correlated with lower response to therapy and disease progression [22]. Parallel to this study, a number of studies have suggested that a two-way communication is established between leukemic cells and endothelial cells in the bone marrow of patients with AML. These two cells support the survival and proliferation of each other through a paracrine pathway, resulting in resistance to chemotherapy. In addition, leukemic cells have autocrine effects on themselves. AML cells express receptors for angiogenic factors, such as VEGFR1 (about 50% of leukemic blasts) and VEGFR2 (about 20% of leukemic blasts); they also secrete angiogenic factors such as vascular endothelial growth factor (VEGF) [23-28]. VEGF produced by leukemic cells is able to activate its receptor on both leukemic and endothelial cells [29]. After binding to its receptor on leukemic cells, VEGF can serve as an autocrine growth factor, conferring protection against apoptosis through increased expression of myeloid cell leukemia 1 (MCL1) (a family member of B-cell lymphoma 2 [BCL2]) [27]. VEGF can also bind to its receptor present on endothelial cells, resulting in the induction of angiogenesis as well as the stimulation of endothelial cells to secrete soluble growth factors including granulocyte colony-stimulating factor (G-CSF), granulocyte-macrophage colony-stimulating factor (GM-CSF), VEGF and interleukin-6 (IL-6), which are important in proliferation and survival of leukemic cells (Fig. 1) [2, 30]. 

In addition to secreted factors, cell-cell interactions between leukemic cells and adhesion molecules on the surface of endothelial cells lead to the development of leukemia [31, 32]. There is a wide variety of receptors on the surface of leukemic cells, including CXCR4, very late activation antigen 4 (VLA4), and CD44. The receptors bind to their ligand on stromal cells, such as endothelial cells, resulting in activation of survival and proliferation pathways in leukemic cells. This allows leukemic cells to become resistant to chemotherapy, a process known as adhesion-mediated drug resistance (CAM-DR) [18, 33]. In the bone marrow, AML cells have been shown to interact with endothelial cells, in which leukemic cells closer to endothelial cells are more resistant to conventional chemotherapy. Based on these, we conclude that endothelial cells protect leukemic cells, and blood vessels serve as a haven for them [20].

The Role of Endothelial Cells after Transplantation

In general, before the transplantation, the number of endothelial cells increases in the peripheral blood and bone marrow of patients with AML; this is associated with relapse and resistant to therapy. Researchers have identified that after HSCT, circulating ECs constantly increase until day 21 [11]. Moreover, studies suggest that after HSCT, endothelial damage may occur directly or indirectly due to various factors, which lead to neovascularization and recruitment of inflammatory cells. Researchers have reported that vasculogenesis involves in the process of new vessel formation after HSCT [11]. Vasculogenesis differs from angiogenesis; it is the process of blood vessel formation occurring by a *de novo* production of endothelial cells from mesodermal cell precursors [34]. The vasculature is directed by allo-reactive donor T cells, vascular injury happens and endothelial biological markers are raised in the peripheral blood of patients [11]. Since after HSCT, occurrence of GVHD depends on neovascularization and endothelial damage, increased markers of EC biology such as von Willebrand factor (vWF) and thrombomodulin in the peripheral blood can be useful in the diagnosis of GVHD [11]. Moreover, during treatment of GVHD, selective inhibition of vasculogenesis process compared with the inhibition of angiogenesis has less undesirable effects; since various physiologic processes such as wound healing and tissue regeneration, depend on angiogenesis [11].

Assessment of Bone Marrow Microvessel Density (BM-MVD) in Patients with AML

**Figure 2:**
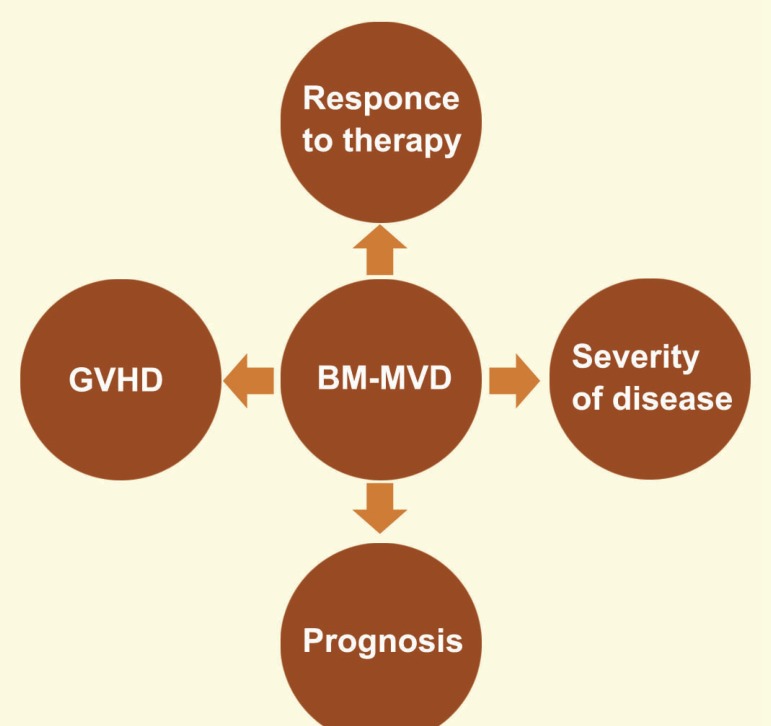
In the bone marrow of patients with AML increased levels of BM-MVD are associated with increase the severity of disease, reduce response to chemotherapy, poor prognosis and increased incidence of graft versus host disease (GVHD

Currently, the bone marrow biopsy is used to assess angiogenesis in patients with AML. After special staining, the vessels are examined for vascularity, the so-called MVD, and morphology. In the bone marrow of patients with AML, there is great evidence for increased angiogenesis and neoangiogenic processes, including increased microvessel density, the presence of hot spots, endothelial sprouts without visible lumina, and branching microvessels with variable morphologies [35]. A number of studies showed that MVD is significantly higher in patients with AML at the time of diagnosis, as compared to healthy controls, and decreases after remission [35-37]. BM-MVD levels are associated with the severity of disease and therapeutic responses. Therefore, increased levels of BM-MVD are related to a poor prognosis in patients with AML [38]. In both complete remission (CR) and non-remission (NR) patients, MVD were significantly higher than control groups at diagnosis. After treatment, MVD shows a significant reduction in the CR patients but still remains high in the NR patients. Therefore, MVD is associated with the response to chemotherapy (Fig 2) [36, 37].

Morphological changes in microvessels, along with quantitative differences in microvessel counts, were observed at the time of diagnosis and after chemotherapy. At the time of diagnosis, endothelial cell sprouts and microvessels, without visible lumina, are dominant in bone marrow samples from patients with AML [35, 36]. However, these morphological characteristics in patients with AML are subject to variation after complete remission; microvessels reach a large size with a visible, dilated, wider and empty lumen, endothelial cell sprouts are completely absent, a reduction in endothelial clusters occur, and microvessels represent a sinusoidal form [35, 36].

After HSCT new vessels formation in the BM are increased in patients with acute GVHD. This neovascularization originates mostly from recipient endothelial cells [39]. In addition, patients with acute GVHD had a considerably higher MVD than patients free of acute GVHD and the angiogenic activity reduce after the GVHD resolves [39]. On the contrary, patients with chronic GVHD did not have a considerably altered MVD and had no impressive angiogenic activity in their BM [39].

Assessment of VEGF in Patients with AML

VEGF is one of the most important positive regulators of angiogenesis the expression of which is induced by hypoxia. It promotes proliferation, migration, and differentiation of endothelial cells, and increases their permeability to plasma proteins [40, 41]. In hematologic malignancies, VEGF stimulates a mitogenic response, and upgrades self-renewal of leukemic cell progenitors [23, 42]. In patients with AML, blast cells permanently produce and secrete VEGF, leading to increased levels of VEGF in bone marrow and serum [43]. VEGF then binds to endothelial or leukemic cells, increases the phosphorylation and transmission of signals into the cells, and upgrades cell proliferation [44]. Since plasma, serum, and bone marrow VEGF levels differ in patients with AML compared to healthy subjects (Table 1) [28], here we review its relationship with outcome of the disease.

**Table 1:** Assessment the levels of VEGF in plasma and bone marrow in various times of the disease in patients with AML

Time	Plasma VEGF level	Bone marrow VEGF level
At time of diagnosis	Increase	Increase
After remission	Decrease	Decrease
After treatment	Decrease	Decrease
Disease relapse	Increase	Increase
GVHD occurrence	Conflicting	Conflicting

Plasma and serum VEGF levels are correlated with platelet counts and total WBCs [45]. Higher plasma VEGF levels are associated with the lower complete remission (CR) rate and shortened survival time [20]. In addition, there is no significant difference in serum VEGF levels between adults and children with AML [46].

Bone marrow VEGF concentrations are directly correlated with BM-MVD [20]. High VEGF levels in the bone marrow of patients with AML are associated with higher WBC counts, bone marrow blast percentage, shorter survival, and disease-free survival time [20]. 

VEGF-C mRNA

**Figure 3:**
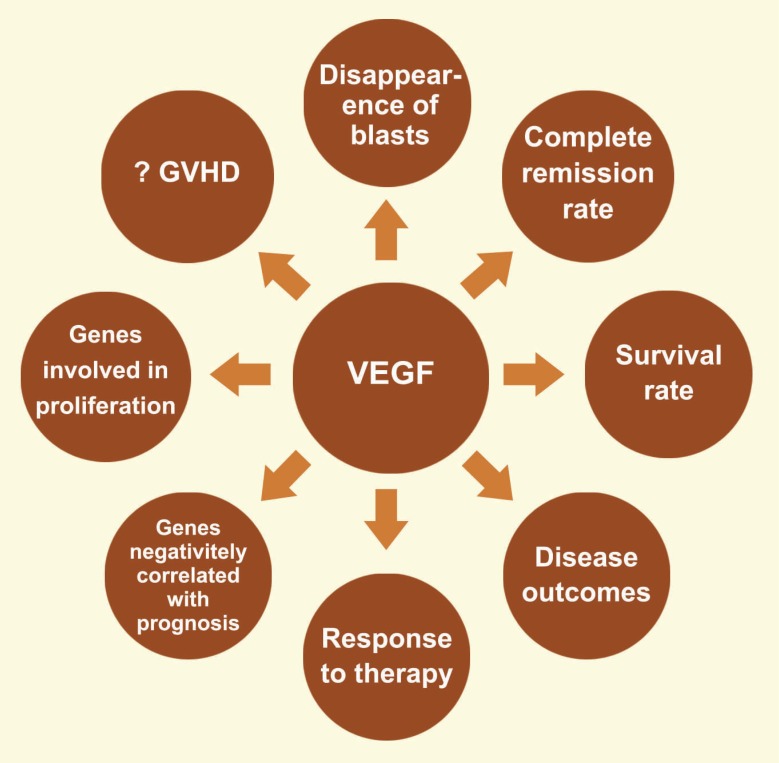
In patients with AML high level of VEGF is associated with slow disappearance of blasts during treatment, lower complete remission rate, reduced survival rate, inferior disease outcomes, and decreased responses to chemotherapy. Also high VEGF levels trigger upregulation of genes involved in proliferation and genes that are negatively correlated with prognosis. In articles, there are very contradictory opinions about the role of VEGF and its effects on graft versus host disease (GVHD) occurrence

There is a lot of evidence that expression of VEGF-C mRNA in leukemic cells significantly alter before and after chemotherapy [47]. Furthermore, VEGF-C released from the endothelial cells is able to increase proliferation and survival rates of AML cells [48]. Immunohistochemical staining showed that higher VEGFC is expressed in patients with AML compared to healthy controls [44]. Because VEGFC expression can predict outcome and prognosis in both pediatric and adult patients with AML, higher expression of VEGFC transcripts are correlated with slow disappearance of blasts during treatment, lower CR rate, reduced overall survival (OS) and event-free survival (EFS), and inferior disease outcomes [49]. High VEGFC levels trigger upregulation of genes involved in proliferation such as p21 protein activated kinase (PAK3), Ras/Rho guanine nucleotide exchange factor 2 (SOS2), extracellular-signal-regulated kinases (ERK), phosphoinositide-dependent kinase-1(PDK1), and protein kinase B (Akt/PKB) (involved in survival) [49-51], as well as, some genes, including ATP-binding cassette family ABCC3 (or MRP3), ABCB9, and ABCC8 that are negatively correlated with prognosis of patients [49]. Furthermore, increased VEGF-C gene expression is accompanied by decreased responses to chemotherapy in both adult and pediatric patients with AML (Fig 3) [20].

After HSCT

There are contradictory opinions about the role of VEGF and its effects on HSCT outcome and GVHD occurrence. Some studies have suggested that high circulating levels of VEGF after HSCT are useful and have a protective effect on occurrence of GVHD [52, 53]. Furthermore, high VEGF levels after HSCT are associated with lower occurrence of GVHD [52, 53], and a tendency towards less-severe acute GVHD [54]. On the other hand, some studies have suggested that VEGF levels are elevated in the bone marrow of patients with acute GVHD [8, 55] and can serve as a helpful endothelial marker [56]. Before HSCT, high VEGF-A level is associated with increased risk of relapse, reduced survival rate and negative prognostic effect on HSCT outcome [56]. Moreover, after HSCT, high VEGF level is accompanied by elevated risks of early complications and non-relapse mortality (NRM) [56].

Assessment of Ang-Tie in Patients with AML

**Figure 4:**
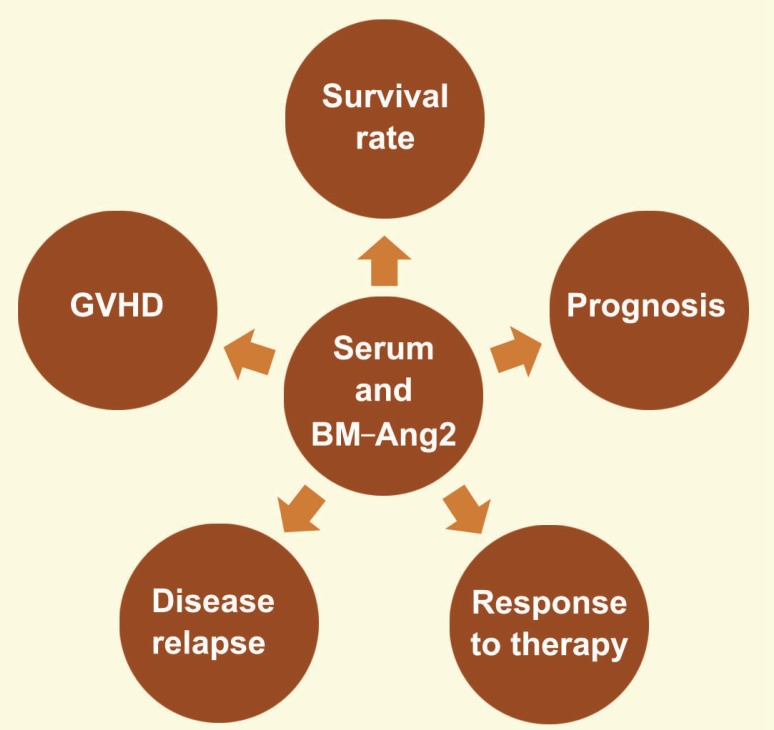
In patients with AML, high levels of Ang-2 in the bone marrow and serum are associated with shorter survival rate, poor and unfavorable prognosis, decreased response to therapy, increased risk of disease relapse and increased incidence of post-transplant GVHD

Angiopoietin and its receptor, Ang-Tie, play an important role in angiogenesis. Ang-1 and Ang-2, although exhibiting similar affinity for their receptor, Tie2, oppose each other [57]. Ang1 binding to Tie2 is thought to inhibit angiogenesis in mature vessels and Ang-2 was shown to be a prognostic factor in AML [58, 59]. In patients with AML, high Ang-2 levels are found in the bone marrow, and expression of the Tie-2 receptor increases on myeloid blasts, which are associated with poor prognosis and shortened survival rate [20]. Blocking the connection between Angs and Tie-2 results in decreased proliferation of myeloid blasts [20]. In patients with AML, serum Ang-1 and Ang-2 levels were shown to be significantly lower and higher, compared with healthy controls and patients with myeloproliferative neoplasms (MPN), respectively [59]. High Ang-2 levels are associated with shorter overall survival, unfavorable prognosis, and decreased response to therapy. Moreover, lower Ang-2 levels was found in patients who achieve complete remission (Fig 4) [59].

During HSCT

Some studies have found that pre-HSCT Ang-2 levels narrowly reveal the extent of neo-angiogenesis in the bone marrow of patients with leukemia [60]; it is also associated with endothelial cell activation and dysfunction [61]. High pre-transplantation Ang-2 levels in serum of patients disclose unfavorable prognosis and low survival rate after allo-HSCT [8, 62]. In addition, Ang-2 was recognized as a potent predictor for time-to-relapse in the setting of HSCT [8, 62]. Some articles have indicated that after HSCT, there is a tendency to increasing Ang2 levels. Moreover, higher post-transplantation Ang2 levels are associated with severe skin and liver GVHD and both higher NRM and lower survival rate [8]. Therefore, measurement of circulating Ang-2 concentrations after HSCT might help physicians to estimate the risk of relapse and individualize the post-transplantation treatments [62].

## DISCUSSION

In this review we have demonstrated that a two-way communication is established between leukemic cells and endothelial cells in the bone marrow of patients with AML. These two cell types support the survival and proliferation of each other through a paracrine pathway and cell-cell interactions, resulting in resistance to chemotherapy and development of leukemia [23-28]. Based on these, we concluded that endothelial cells protect leukemic cells, that blood vessels serve as a haven for them, and that VEGF plays an important role [20]. Parallel to our study, one study suggests that the evaluation of bone marrow aspirates in patients with AML can show both cytoplasmic and extracellular VEGF expression (*in vivo*). However, when the cells were cultured for two days, the levels of VEGF in patients with AML were similar to those in normal controls (*in vitro*). Due to this discrepancy, we concluded that angiogenesis is not an intrinsic property of patients with AML and is likely induced by microenvironmental elements [63]. 

We also showed that MVD is significantly higher in patients with AML at the time of diagnosis, as compared to healthy controls, and decreases after remission [35-37]. In addition BM-MVD levels are associated with the severity of the disease and therapeutic response [38]. Unlike the findings of this review, Teresa Padro and co-workers [35] reported that MVD increases in patients with AML at the time of diagnosis and decreases on day 16 of induction chemotherapy. However, higher microvessel counts were observed at the time of complete remission. They believed that direct or indirect chemotherapy effects on endothelial cells can explain different alterations in microvessel density during the treatment. In response to an unknown angiogenic inducer, endothelial cells can become activated from their dormant state and proliferate rapidly. Chemotherapy agents may directly influence these rapidly proliferating cells, and also induce apoptosis in leukemic blasts, resulting in disappearance of indirect survival signals in endothelial cells [35]. Therefore, a decreased microvessel density is detected on day 16 of induction chemotherapy. Recovery of endothelial cells from cytotoxic chemotherapy leads to increased microvessel counts at the time of complete remission [35].

Another finding of this study was that plasma, serum, and bone marrow VEGF levels in patients with AML were high at the time of diagnosis and dramatically decreased after treatment (Table 1). In contrary to this statement, in one study, serum VEGF levels were demonstrated to increase after treatment in both AML complete remission (CR) and AML non-remission (NR) patients. However, a significant decrease in bone marrow VEGF levels was found in both groups of patients after treatment [37]. 

As mentioned earlier, there are conflicting views on the impact of VEGF on transplantation consequence. Controversial results in the literatures can be described by dissimilar study designs, specimen collection at various times after HSCT, and lack of precise VEGF reference values for pediatric patients and plasma samples [8]. Some differences among the results of the studies were due to the use of serum instead of plasma [45]. Platelets and WBCs release high levels of VEGF into serum during the clotting process. Therefore, it is feasible to use plasma rather than serum to analyze angiogenic factors [45].

Finally, it is important to note that after HSCT an increase in the levels of angiogenic factors involved in repair/regeneration (such as VEGF-A, -C, -D), might improve transplant consequences [61]. Conversely, patients with high levels of angiogenic factors, which facilitate damage/inflammation (such as angiopoietin-2), exhibit poor outcomes in terms of HSCT [61]. Furthermore, the effect of an angiogenic factor should not be interpreted separately and other factors participating to the angiogenic milieu must also be considered [61]. In this regard, Porkholm has shown that higher angiopoietin-2 levels are accompanied by higher non-relapse mortality and, if VEGF levels increase at the same time, patients’ survival reduces even more than before [8]. Additionally VEGF, found in the blood circulation, is normally removed by serum protease activity. Whenever GVHD occurs, large amounts of VEGF release into blood circulation in response to endothelial injury. The serum protease activity is unable to overcome such extensive amounts of VEGF. As a result, the increased circulating VEGF levels could be seen [64-66]. So, it is likely that the increased VEGF level, which observed in patients with GVHD, has reactive mode and have no direct influence on prognosis.
